# The Blowfly *Chrysomya megacephala* as a Vector of Pathogens Associated with Infectious Diseases

**DOI:** 10.3390/pathogens15030300

**Published:** 2026-03-10

**Authors:** César Valverde-Castro, Alba Luz Peralta-Botello, Maria Teresa Mojica

**Affiliations:** Grupo de Investigación en Medicina Tropical (CIMET), Universidad del Magdalena, Santa Marta 470003, Colombia; albaperaltalb@unimagdalena.edu.co (A.L.P.-B.); mtmojica@unimagdalena.edu.co (M.T.M.)

**Keywords:** 16S, Calliphoridae, Colombia, forests, insect vectors, MinION, nanopore sequencing, public health, rural, urban

## Abstract

*Chrysomya megacephala* is a synanthropic fly with a high potential to act as a mechanical vector of pathogenic bacteria, surpassing *Musca domestica* in both bacterial load and diversity. Native to Asia and Africa, it has become a cosmopolitan species, successfully adapting to a wide range of environments, including natural ecosystems. In Colombia, studies on its role as a vector are limited and have largely relied on traditional culturing methods. This study aimed to characterize the pathogenic bacterial microbiota associated with *C. megacephala* using 16S rRNA gene sequencing in urban, rural, and forest settings of a coastal tourist city. Flies were collected using Van Someren Rydon traps with attractants and sterile materials. Bacterial identification was performed through Oxford Nanopore MinION sequencing (Manufactured by Oxford Nanopore Technologies, Oxford, UK). A total of 49 bacterial species were identified, with urban environments showing the highest taxonomic richness. The forest environment was characterized by a highly dominant community structure, led by *Vagococcus carniphilus*. Notably, 20 bacterial species of public health relevance were detected, including *Clostridium botulinum*, *Clostridium perfringens*, *Ignatzschineria ureiclastica*, *Escherichia coli*, and *Streptococcus agalactiae*. These findings indicate that bacterial community composition varies by environment and underscore the potential role of *C. megacephala* as a mechanical vector, highlighting the importance of surveillance for its public health implications.

## 1. Introduction

*Chrysomya megacephala* (Fabricius, 1794) has been categorized as a synanthropic species because of its close association with human settlements, and like *Musca domestica* Linnaeus, 1758, it has been widely recognized as a mechanical vector of pathogens, largely owing to its feeding and oviposition habits [1]. Various studies have shown that *C. megacephala* poses a greater epidemiological risk. This species was reported to carry bacteria in 87.7% of the analyzed samples, whereas it was found in 66.2% of the *M. domestica* samples, indicating a greater diversity of bacterial species [1,2]. Additionally, in urban environments, *C. megacephala* was found to be not only more abundant than *M. domestica* but also to exhibit bacterial positivity rates ranging from 96.4% to 100%, harboring bacterial loads up to 11–12-fold higher [2]. Although both species constitute more than 50% of their microbiome, *C. megacephala* presents greater diversity and a greater load of pathogenic bacteria, including clinically relevant species such as *Escherichia coli*, *Enterobacter cloacae*, and *Pseudomonas* spp. [3].

*Chrysomya megacephala* belongs to the family Calliphoridae (Diptera) and is commonly known as the oriental latrine fly. It is an exotic species that is native to Asia and Africa and was introduced into South America in the late 1970s [4]. Its remarkable dispersal ability, high fecundity, and ecological adaptability have allowed it to be rapidly established in various environments [5]. It is now considered a cosmopolitan species and is currently widely distributed across the globe [6,7]. It is a necrophagous and saprophagous species that is strongly associated with urban environments but is also capable of colonizing natural habitats. The presence of this species has been recorded in fragments of tropical dry forest—an ecosystem with minimal human intervention—indicating that it does not rely exclusively on urban or degraded environments to thrive [5].

In Colombia, research on bacteria associated with synanthropic flies has been limited and has focused mostly on *M. domestica* [8,9]. A single study including multiple fly species reported that *C. megacephala* had the highest mechanical vector risk index (MVRI), indicating a high capacity to transport pathogenic bacteria from contaminated sources to surfaces in contact with humans [10].

All of these studies employed conventional culturing techniques, representing a significant limitation, as it is estimated that only ~1% of the bacterial microbiome is cultivable. In contrast, next-generation sequencing (NGS) can characterize the entire microbial diversity of a sample and compare it to known nucleotide sequences in databases, enabling the identification of all sequenced organisms [11]. Despite methodological constraints, seven different bacterial species have been identified in *C. megacephala*, including *Escherichia coli*, *Providencia rettgeri*, *Pasteurella pneumotropica*, *Kluyvera* sp., *Serratia odorifera*, *Chryseobacterium meningosepticum*, and *Enterobacter sakazakii*, highlighting the underestimated epidemiological relevance of this species in the Colombian context [10].

Recent studies using metagenomic techniques have shown that the external surfaces of this fly—particularly the legs and wings—harbor a greater quantity and diversity of bacteria than its internal structures, including numerous clinically important pathogens [3]. This elevated microbial load is facilitated by morphological structures on its exoskeleton, such as microvilli, setae, and hairs, which enhance microbial retention [9].

Given this evidence, the present study aims to identify the pathogenic bacteria associated with *C. megacephala* in a coastal tourist city with a high influx of national and international visitors by examining its external microbiota in urban, rural, and forest environments through 16S rRNA gene sequencing. This approach will not only expand the knowledge of its vector potential across gradients of anthropogenic disturbance but also provide critical insights into the health risks posed by this species in global tourist settings, where interactions between humans and synanthropic vectors may facilitate the spread of emerging pathogens.

## 2. Materials and Methods

### 2.1. Study Area and Fly Collection

**Figure 1:**
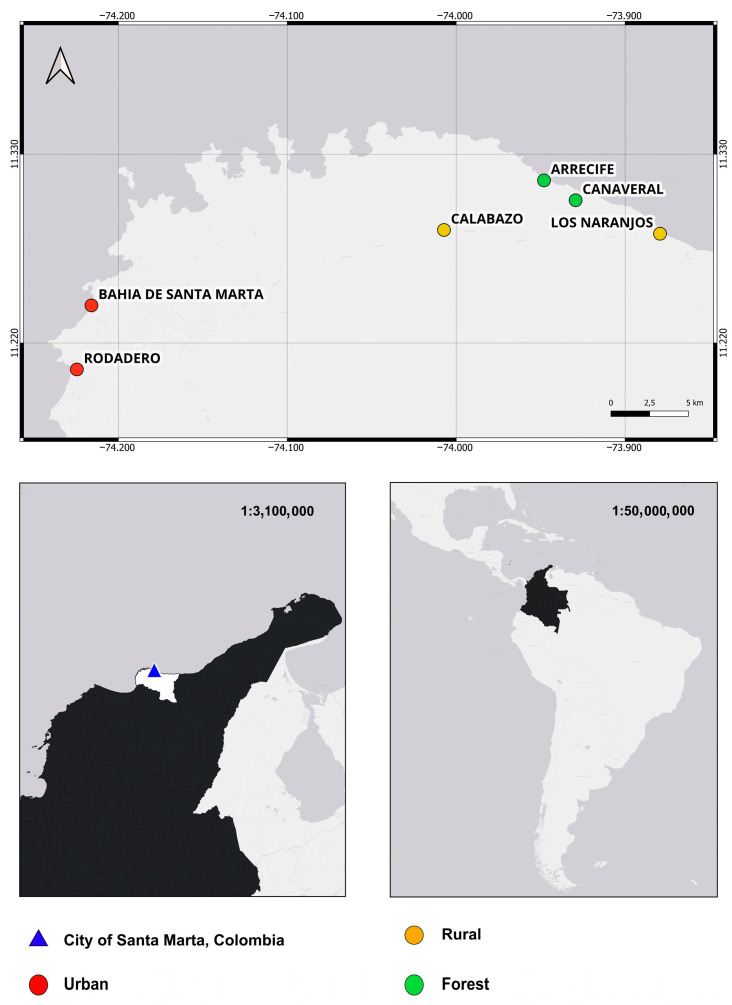
Map of sampling points in Santa Marta, Colombia. The location of Colombia is highlighted in black.

The study was conducted in the northern region of South America, along the Caribbean coast, in the city of Santa Marta, Colombia (Figure 1). This city is one of the main tourist destinations in the Caribbean region and is surrounded by beaches of high recreational and ecological value that attract thousands of national and international visitors year-round. It has a tropical dry climate, with average annual temperatures between 27 and 30 °C and annual rainfall ranging from 500 to 1000 mm. Six zones with distinct environmental characteristics were selected. The urban zones of El Rodadero and Bahía de Santa Marta are located within the city core and are characterized by high population density, intense tourist activity, and extensive infrastructure. In contrast, the rural zones of Calabazo and Los Naranjos feature a landscape dominated by secondary vegetation and mixed land use combining agriculture and ecotourism, with scattered, low-density populations. Finally, the areas of Cañaveral and Arrecife represent forested zones located within Tayrona National Natural Park, a tropical dry forest ecosystem with high biological diversity and varying degrees of conservation. Together, these sites represent an ecological gradient with marked variations in vegetation cover, anthropogenic disturbance, and the availability of decomposing organic matter.

Field collection was carried out in January, April, July, and October 2024. Each sampling effort lasted 12 h, during which three Van Someren Rydon traps were installed 50 m apart at a height of 1.5 m. The traps were baited with decomposing fish, fermented fruit, and human feces to simulate the diversity of organic matter present in these environments. The attractants were placed in plastic containers covered with tulle mesh to prevent direct contact between the flies and the bait. Inside each trap, sterile muslin fabric mesh was used to capture live samples, which were euthanized by freezing at −80 °C to avoid cross-contamination. Additionally, 1.5 mL Eppendorf tubes containing saline solution were placed near the traps as environmental controls.

The collected and frozen flies were individually placed into Eppendorf tubes with saline solution for external washing. To dislodge surface bacteria, each sample was vortexed for 5 min at 1000 rpm. After this procedure, the flies were removed and taxonomically identified using the Whitworth key [12] to confirm the identity of *Chrysomya megacephala*.

For each environment (urban, rural, or forest), external washes from ten flies were combined into a single composite sample. Each sample was then centrifuged at 10,000 rpm for 5 min to concentrate the bacteria at the bottom of the tube, from which bacterial DNA was extracted. This procedure resulted in one sequenced sample per environment.

### 2.2. Molecular Procedures

Genomic DNA was extracted using the HiPurA Multi-Sample HiGenoMB Kit (HiMedia Laboratories Pvt, Ltd., Maharashtra, India), with key modifications to the manufacturer’s protocol. Microcentrifuge tubes containing the bacterial suspension were first centrifuged at 12,000× *g* for 8 min at 4 °C. The resulting pellet was resuspended in 200 μL of lysozyme solution (45 mg/mL) and incubated at 37 °C for 40 min in a thermomixer at 300 rpm. Next, 20 μL of proteinase K (20 mg/mL) and 200 μL of lysis solution (C1) were added, followed by vortexing for 10 s and incubation at 55 °C for 30 min. The extracted DNA was eluted in 50 μL of DNase- and pyrogen-free water and subsequently stored at −80 °C. DNA concentration and quality were assessed using a Qubit 3 fluorometer (Invitrogen, Life Technologies Holdings Pte Ltd., Singapore), and purity was evaluated via the A260/A280 ratio [13].

Amplification of the 16S rRNA gene was performed using TaKaRa LA Taq polymerase [14] with the universal primers 27F (5′-AGAGTTTGATCMTGGCTCAG-3′) and 1492R (5′-GGTTACCTTGTTACGACTT-3′) [15]. PCRs were conducted using 4 μL of extracted DNA under the following cycling conditions: initial denaturation at 94 °C for 2 min, followed by 30 cycles of 94 °C for 30 s, 55 °C for 30 s, and 72 °C for 90 s, with a final extension at 72 °C for 10 min.

Library preparation was performed using the Native Barcode Kit 96 V14 (SQK-NBD114.96), and sequencing was carried out on a MinION FLO-MIN114 flow cell (R10 version) at the Centro de Genética y Biología Molecular, Universidad del Magdalena.

### 2.3. Bioinformatic Analyses

The raw data were processed via Dorado v0.9.1 for basecalling, employing the SUP (super accuracy basecalling) algorithm with the dna_r10.4.1_e8.2_400 bps_sup@v5.0.0 model, retaining reads with a Phred quality score ≥ 10. Demultiplexing was performed under strict parameters, retaining only reads with valid barcodes at both ends. Read quality was assessed using NanoPlot v1.44.1 [16], and adapters and chimeric sequences were removed using Porechop v0.2.4 [17]. The tool fastp v0.24.0 [18] was used to retain reads between 1400 and 1700 bp, and Cutadapt v3.5 [19] was applied to trim primers with a 25% error tolerance. Reads containing internal primers were discarded. Final quality checks were performed using NanoPlot and MultiQC v1.24.1 [20].

Taxonomic classification and read curation were conducted via the PRONAME pipeline [21] with the SILVA 138 SSURef N99 database [22]. The reads were clustered into OTUs using VSEARCH v2.22.1 [23] at a 98.7% similarity threshold [24]. Consensus sequences (CSs) were generated by selecting centroids and 100 random reads per cluster using Seqkit v2.3.0 [25], followed by error correction using Medaka v2.0.1. Chimeric sequences were detected with VSEARCH using SILVA as a reference.

After contaminant removal, a second validation of the consensus sequences was performed via BLASTn v2.15.0 [26] against the NCBI nonredundant database, considering alignments valid if they met the following criteria: identity ≥ 98.7%, coverage ≥ 99%, E value ≤ 0, and bit score ≥ 50 [24]. The final results were exported in a QIIME2-compatible format for visualization via QIIME2 View (https://view.qiime2.org/).

### 2.4. Bacterial Diversity Analyses

Taxa detected in negative controls, both laboratory and environmental, as well as reads that could not be classified at the species level, were excluded from subsequent analyses. Only low-abundance environmental taxa were identified in the controls, which were later removed to minimize the risk of potential contamination. Analyses were conducted using relative abundance data. Diversity indices, including the Shannon index (H’) and the dominance index (D), were calculated using PAST version 4.12 [27]. Because only one composite sample was obtained for each environment, these indices were used exclusively for descriptive and exploratory purposes and were not interpreted as estimates of within-environment variability. Finally, a correspondence analysis (CA) was performed using only medically and veterinary-relevant bacterial species to explore and visualize specific associations between taxa and the environments in which *C. megacephala* was collected.

## 3. Results

A total of 49 bacterial species were identified. *Vagococcus carniphilus* was the most abundant (16,596 reads; 57.65%) and was detected across all environments, with the highest number of reads recorded in the forest (7542 reads) and urban (6479 reads) environments. *Streptococcus infantarius* (4916 reads; 17.08%) and *Weissella cibaria* (3185 reads; 11.06%) were subsequently detected and were primarily associated with the urban environment, where we found their highest read counts (Table 1 and Table S1). In the urban environment, the bacterial community consisted of 32 detected taxa, with an even community structure reflected in its diversity metrics (H’ = 1.63; D = 0.27). The rural environment contained 30 taxa, and its community exhibited intermediate levels of diversity and evenness (H’ = 1.49; D = 0.39). The forest environment also presented 30 taxa; however, its community structure was characterized by the predominance of a single species representing more than 90% of the total reads, which was associated with lower overall diversity (H’ = 0.56; D = 0.82).

The identification of medically and veterinary-relevant bacteria on the external surface of *C. megacephala* underscores the potential of this species to act as a mechanical vector of microorganisms that may impact human and animal health. To explore the clinical relevance of the identified taxa, a literature review was conducted, and the findings are summarized in Table 2. Several of the detected bacterial species, although commonly found in the environment or in food products, have also been implicated in opportunistic infections and serious diseases. These results highlight the importance of considering the environment in which *C. megacephala* is found when assessing its potential role in the transmission of pathogenic bacteria.

**Figure 2:**
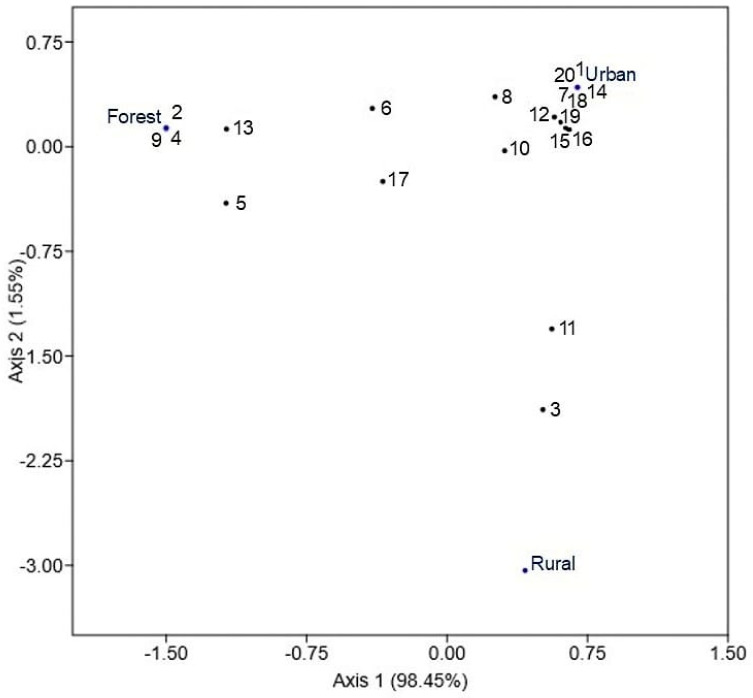
Correspondence Analysis (CA) between bacteria of medical-veterinary importance and environments. The codes represent the 20 species of bacteria (see Table 2).

The correspondence analysis (CA) enabled visualization of associations between medically and veterinary-relevant bacterial taxa and the sampled environments (Figure 2). The results revealed that several pathogenic species were preferentially associated with the urban environment, whereas others clustered around forest samples. Moreover, the rural environment presented a distinct bacterial profile, separated from the urban and forest environments in the ordination space. The first axis explained 98.45% of the total variability, capturing most of the differences in the bacterial‒environment associations.

Overall, these results demonstrate that *C. megacephala* harbors a diverse bacterial community on its external surface, including taxa of medical and veterinary importance, and that these communities vary depending on the environment. These findings raise new questions regarding the potential role of this species in the dispersal of microorganisms across different ecological contexts. While the presence of medically relevant bacteria on the fly’s external surfaces supports the hypothesis of a possible role as a mechanical vector, it is important to note that this study does not directly evaluate transmission efficiency or clinical outcomes associated with infection.

## 4. Discussion

*Chrysomya megacephala* is a synanthropic species strongly associated with environments rich in decomposing organic matter, such as carcasses, excreta, and household waste [1,2]. In this study, the bacterial community associated with the surface of *C. megacephala* was dominated by the phylum Firmicutes, followed by notable representatives of Proteobacteria and Actinobacteria, reflecting a microbial structure shaped by the insect’s ecological environment and food sources [48]. These results are consistent with previous findings reporting high microbial diversity on both the external surface and internal organs of *C. megacephala* and *M. domestica*, including numerous genera with pathogenic potential [3].

One of the most notable findings of this study was the marked prevalence of *Va. carniphilus*, a species previously documented in raw meat, animal intestines, and fermentative substrates [49], as well as in farmed fish, where it has shown pathogenic potential by being associated with skin lesions, hemorrhages, septicemia, and significant tissue damage, highlighting its veterinary importance and zoonotic potential [44]. In our dataset, *Va. carniphilus* was consistently detected in all sampled environments, with a higher number of reads in the forest environment, followed by the urban and rural environments, suggesting a remarkable ecological adaptability of this bacterium and raising relevant questions about its reservoirs and transmission routes. Various studies indicate that *C. megacephala*, due to its necrophagous habits, regularly comes into contact with wildlife carcasses, feces, and other decomposing organic materials [1,2,7], which likely facilitates the acquisition of this bacterium on the external surface of the fly.

The consistent detection of *Va. carniphilus* in all samples also suggests a possible stable ecological association with *C. megacephala*, possibly of a facultative symbiotic nature. As a necrophagous species, *C. megacephala* relies on carrion for oviposition and larval development; the presence of *Va. carniphilus* could accelerate the initial decomposition of tissues, increasing nutrient availability for the larvae and thus favoring their survival and growth. In turn, the bacterium could benefit from its dispersion to new decomposing substrates through the fly’s feeding and reproductive behavior.

*Streptococcus infantarius* was detected mainly in the urban environment, which may be attributed to its intestinal origin and its release into the surroundings through wastewater, household waste, and decomposing organic matter; in this context, recurrent fecal contamination and inadequate waste management create moist, nutrient-rich microhabitats that favor the persistence of non-spore-forming bacteria such as this species [50]. Its ability to survive outside the host and cause opportunistic infections, such as endocarditis and bacteremia, confers medical relevance, and the increasing frequency of its clinical isolation demands greater recognition in microbiological diagnostics, as well as stricter public health surveillance [43]. Although no reports directly associate flies with *S. infantarius*, their potential role as passive vectors in the dispersion of intestinal bacteria represents an ecological aspect that warrants further investigation. Likewise, the identification of *S. agalactiae*, a well-known human pathogen implicated in infections such as septicemia, meningitis, and pneumonia, underscores the risk of indirect transmission from contaminated surfaces [41]. These findings are in line with previous reports of potentially pathogenic streptococci isolated from flies collected in Colombian hospitals, further supporting the hypothesis of cross-transmission via nontraditional vectors [9].

In this study, two species of the genus *Weissella* were mainly detected in the urban environment: *We. cibaria* and *We. confusa*. The presence of *We. cibaria* is consistent with previous studies that have documented its external occurrence in synanthropic flies such as *M. domestica* and *C. megacephala* in Brazil, the United States, and Singapore [3]. In contrast, *We. confusa* had not previously been reported on the surface of these insects; however, it has been identified in the gut of *M. domestica* larvae fed sugar-rich diets, suggesting an ecological affinity for fermentative environments [51]. Both species are naturally associated with carbohydrate-rich substrates, such as fermented fruits and organic waste, similar to the attractants used in this study. In addition to their involvement in food fermentation processes, *We. cibaria* has been widely recognized for its probiotic properties [46,52], while both it and *We. confusa* include strains with opportunistic potential in clinical contexts, particularly in immunocompromised individuals [46,47]. The high prevalence of these bacteria in urban environments could be explained by the abundance of organic waste and the intense interaction of synanthropic flies with these substrates, which facilitates their acquisition and dispersion in the environment.

In parallel, the identification of *I. ureiclastica*, a genus associated with myiasis and bacteremia secondary to dipteran larval infestations [35], is clinically relevant. Although this study focused on adult flies, the persistence of this genus throughout the dipteran life cycle suggests potential risk in urban areas where contact between flies, open wounds, and organic waste is prevalent [2,53]. Therefore, controlling urban fly populations not only prevents infestations but also may serve as an indirect strategy to reduce the spread of emerging bacterial pathogens.

The detection of *Cl. botulinum* in flies from the forest environment can be explained by the specific ecological conditions of this habitat, characterized by moist soils, abundant organic matter, and active processes of animal decomposition that favor the persistence and germination of its spores [29]. This microorganism holds great medical relevance due to its ability to produce botulinum toxin, one of the most potent known, which causes botulism, a severe neuroparalytic disease that, although infrequent in humans, leads to high mortality among wildlife, particularly birds [29,54]. In this context, necrophagous flies play a key ecological role as mechanical vectors, since by developing on decomposing organic matter, they can acquire and transport spores to new environments, contributing to the pathogen’s dispersion and the maintenance of its natural cycle within the forest ecosystem [29].

Although in lower abundance, clinically relevant species such as *M. morganii*, *Cl. perfringens*, and *E. coli* have also been identified, all of which are associated with opportunistic infections and antimicrobial resistance [30,31,33,40].

Previous studies in Colombia [10] reported the presence of multidrug-resistant enterobacteria in flies collected from hospitals and urban markets, which may explain the detection of these bacteria in our samples, particularly in densely populated areas. The ability of *C. megacephala* to act as a temporary reservoir for these pathogens underscores its importance in urban microbial ecology.

The results confirm that *C. megacephala* can harbor clinically relevant bacterial species, including opportunistic bacteria, especially in tropical regions where it is abundant, synanthropic, and commonly found in human environments [3]. The consistency of these results with studies conducted in other geographic regions suggests that ecological factors, such as the high availability of organic waste, frequent contact with excreta, and unregulated urbanization, are common drivers shaping its microbiome. It is important to note that although some of the identified taxa have been associated with human diseases, their pathogenicity largely depends on the host context, as in the case of immunocompromised individuals or those with underlying conditions.

This study provides an initial characterization of the external bacterial microbiota of *C. megacephala* in different environments within a tourist city on the Colombian Caribbean coast, supported by next-generation sequencing and bioinformatic procedures that incorporated confidence thresholds and quality filters to maximize the taxonomic accuracy of 16S rRNA. However, it is important to acknowledge that, by working with a single composite sample per environment, the analyses focus on characterizing the DNA of both live and dead bacteria rather than estimating the presence of viable and infectious propagules of the bacteria within each environment. Likewise, our approach focused exclusively on the external microbiota, as the main objective was to identify the taxa present on the surface of the flies and discuss their potential mechanical transmission. Future studies could complement these findings by including biological replicates, analysis of the internal microbiome, assessments of viability, or studies on the dispersal of live and infectious forms of all detected pathogens—factors that go beyond the scope of this study but represent opportunities to deepen the understanding of the microbial ecology of this fly species.

## 5. Conclusions

Considering these findings, the potential role of *C. megacephala* as a mechanical vector of pathogens is further supported, highlighting its possible involvement in the indirect transmission of infectious diseases in urban environments. The identified bacteria not only pose immediate risks through contamination of food and surfaces but also may serve as indicators of the environmental circulation of opportunistic and emerging microorganisms. The inclusion of *C. megacephala* in disease surveillance programs transmitted or associated with vectors is solidly grounded, considering its ability to act as a mechanical carrier of pathogenic and opportunistic bacteria. To maximize its epidemiological usefulness, it is recommended to focus such surveillance on high sanitary and ecological risk environments, such as food markets, landfills, tourist beaches, and peri-hospital areas, where the interaction between humans, waste, and these mechanical vectors is particularly high.

## Figures and Tables

**Table 1:** Bacteria detected from the outer surface of *Chrysomya megacephala*, in Santa Marta *.

Taxa	Urban	Rural	Forest	Total
	No (%)	No (%)	No (%)	No (%)
*Asaia bogorensis*	21 (0.13)	0 (0)	0 (0)	21 (0.07)
*Clostridium botulinum*	0 (0)	0 (0)	1 (0.01)	1 (0)
*Clostridium perfringens*	2 (0.01)	4 (0.09)	0 (0)	6 (0.02)
*Clostridium* sp.	0 (0)	0 (0)	4 (0.05)	4 (0.01)
*Erysipelothrix rhusiopathiae*	0 (0)	1 (0.02)	5 (0.06)	6 (0.02)
*Escherichia coli*	1 (0.01)	0 (0)	1 (0.01)	2 (0.01)
*Hathewaya limosa*	1 (0.01)	0 (0)	0 (0)	1 (0)
*Ignatzschineria ureiclastica*	362 (2.23)	1 (0.02)	92 (1.1)	455 (1.58)
*Lactobacillus gasseri*	0 (0)	0 (0)	2 (0.02)	2 (0.01)
*Lactococcus lactis*	154 (0.95)	25 (0.59)	35 (0.42)	214 (0.74)
*Leuconostoc pseudomesenteroides*	935 (5.77)	60 (1.41)	54 (0.65)	1049 (3.64)
*Morganella morganii*	11 (0.07)	1 (0.02)	69 (0.83)	81 (0.28)
*Pseudolactococcus raffinolactis*	1 (0.01)	1 (0.02)	0	2 (0.01)
*Streptococcus agalactiae*	343 (2.12)	3 (0.07)	0 (0)	346 (1.2)
*Streptococcus equinus*	83 (0.51)	8 (0.19)	1 (0.01)	92 (0.32)
*Streptococcus infantarius*	4406 (27.19)	405 (9.53)	105 (1.26)	4916 (17.08)
*Vagococcus carniphilus*	6479 (39.98)	2575 (60.59)	7542 (90.54)	16,596 (57.65)
*Veillonella dispar*	7 (0.04)	0 (0)	0 (0)	7 (0.02)
*Weissella cibaria*	2858 (17.64)	220 (5.18)	107 (1.28)	3185 (11.06)
*Weissella confusa*	2 (0.01)	0 (0)	0 (0)	2 (0.01)
Other species **	540 (3.33)	946 (22.26)	312 (3.75)	1798 (6.25)
Total	16,206 (100)	4250 (100)	8330 (100)	28,786 (100)

* No: number of reads assigned, % relative abundance by environment, ** bacteria without medical importance.

**Table 2:** Clinically relevant or opportunistic bacterial species associated with *Chrysomya megacephala* *.

Code	Taxa	Environment	Association
1	*As. bogorensis*	U	Associated with bacteremia in immunocompromised patients [28].
2	*Cl. botulinum*	F	Associated with severe neurotoxicity (botulism) in humans and animals [29].
3	*Cl. perfringens*	U, R	Associated with systemic infections such as gas gangrene and enterotoxemia [30].
4	*Clostridium* sp.	F	Some species are associated with human and veterinary diseases [31].
5	*Er. rhusiopathiae*	R, F	Associated with cutaneous and systemic infections in humans [32].
6	*E. coli*	U, F	Associated with gastrointestinal, urinary, and systemic infections in humans and fish [33].
7	*H. limosa*	U	Associated with empyema and other human infections [34].
8	*I. ureiclastica*	U, R, F	Associated with bacteremia in myiasis-infested wounds [35].
9	*Lb. gasseri*	F	Associated with infections in immunocompromised patients [36].
10	*Lc. lactis*	U, R, F	Associated with opportunistic infections in humans [37].
11	*Ps. raffinolactis*	U, R	Associated with mandibular osteomyelitis in ruminants [38].
12	*Le. pseudomesenteroides*	U, R, F	Associated with bacteremia in immunocompromised patients [39].
13	*M. morganii*	U, R, F	Associated with urinary and systemic infections in humans [40].
14	*S. agalactiae*	U, R	Associated with sepsis, pneumonia, and meningitis in humans [41].
15	*S. equinus*	U, R, F	Associated with bovine mastitis and septicemia [42].
16	*S. infantarius*	U, R, F	Associated with bacteremia, endocarditis, and musculoskeletal infections [43].
17	*Va. carniphilus*	U, R, F	Associated with skin lesions and hemorrhages in fish [44].
18	*Ve. dispar*	U	Associated with severe human infections [45].
19	*We. cibaria*	U, R, F	Associated with opportunistic infections in humans [46].
20	*We. confusa*	U	Associated with bacteremia, endocarditis, and abscesses in humans [47].

* U: urban, R: rural, F: forest.

## Data Availability

The FASTQ files have been deposited in the Sequence Read Archive (SRA) databases (https://www.ncbi.nlm.nih.gov/sra, accessed on: 15 December 2025) under BioProject PRJNA1292279.

## Supplementary Materials

The following supporting information can be downloaded at: https://www.mdpi.com/article/10.3390/pathogens15030300/s1, Table S1: Total bacteria isolated from the outer surface of *Chrysomya megacephala* in urban, rural, and forest environments in Santa Marta.
