# Supplementary material for: The Blowfly Chrysomya megacephala as a Vector of Pathogens Associated with Infectious Diseases
Source: Pathogens. 2026 Mar 10;15(3):300. doi: 10.3390/pathogens15030300 (PMC13029238; doi:10.3390/pathogens15030300)
Supplement: Supplementary file 1 [file pathogens-15-00300-s001.zip › pathogens-4024447-supplementary.pdf]

Table S1. Total bacteria isolated from the outer surface of *Chrysomya megacephala* in urban, rural, and forest environments in Santa Marta

Taxa	Urban	Rural	Forest	Total
	No (%)	No (%)	No (%)	No (%)
Acinetobacter nectaris	109 (0.67)	1 (0.02)	22 (0.26)	132 (0.46)
Asaia bogorensis	21 (0.13)	0 (0)	0 (0)	21 (0.07)
Bacteroides xylanisolvens	12 (0.07)	2 (0.05)	0 (0)	14 (0.05)
Brochothrix thermosphacta	0 (0)	0 (0)	2 (0.02)	2 (0.01)
Candidatus Kinetoplastibacterium sp.	0 (0)	0 (0)	22 (0.26)	22 (0.08)
Catenibacterium mitsuokai	1 (0.01)	262 (6.16)	0 (0)	263 (80.91)
Clostridium botulinum	0 (0)	0 (0)	1 (0.01)	1 (0)
Clostridium perfringens	2 (0.01)	4 (0.09)	0 (0)	6 (0.02)
Clostridium sp.	0 (0)	0 (0)	4 (0.05)	4 (0.01)
Collinsella stercoris	0 (0)	4 (0.09)	0 (0)	4 (0.01)
Dorea formicigenerans	2 (0.01)	53 (1.25)	0 (0)	55 (0.19)
Enterococcus termitis	1 (0.01)	6 (0.14)	18 (0.22)	25 (0.09)
Erysipelothrix rhusiopathiae	0 (0)	1 (0.02)	5 (0.06)	6 (0.02)
Escherichia coli	1 (0.01)	0 (0)	1 (0.01)	2 (0.01)
Faecalitalea cylindroides	0 (0)	0 (0)	1 (0.01)	1 (0)
Hathewaya limosa	1 (0.01)	0 (0)	0 (0)	1 (0)
Ignatzschineria ureiclastica	362 (2.23)	1 (0.02)	92 (1.1)	455 (1.58)
Lactobacillus animalis	0 (0)	4 (0.09)	0 (0)	4 (0.01)
Lactobacillus brevis	90 (0.56)	0 (0)	0 (0)	90 (0.31)
Lactobacillus floricola	153 (0.94)	0 (0)	20 (0.24)	173 (0.6)
Lactobacillus gasseri	0 (0)	0 (0)	2 (0.02)	2 (0.01)
Lactobacillus helveticus	0 (0)	0 (0)	4 (0.05)	4 (0.01)
Lactobacillus kunkeei	0 (0)	0 (0)	4 (0.05)	4 (0.01)
Lactobacillus pontis	0 (0)	5 (0.12)	1 (0.01)	6 (0.02)
Lactobacillus sakei	30 (0.19)	449 (10.56)	0 (0)	479 (1.66)
Lactococcus lactis	154 (0.95)	25 (0.59)	35 (0.42)	214 (0.74)
Leuconostoc pseudomesenteroides	935 (5.77)	60 (1.41)	54 (0.65)	1049 (3.64)
Ligilactobacillus ruminis	1 (0.01)	1 (0.02)	0 (0)	2 (0.01)
Limosilactobacillus reuteri	4 (0.02)	32 (0.75)	6 (0.07)	42 (0.15)
Lonsdalea britannica	0 (0)	0 (0)	18 (0.22)	18 (0.06)
Morganella morganii	11 (0.07)	1 (0.02)	69 (0.83)	81 (0.28)
Neokomagataea thailandica	2 (0.01)	0 (0)	9 (0.11)	11 (0.04)
Olsenella sp.	0 (0)	37 (0.87)	0 (0)	37 (0.13)
Parolsenella catena	0 (0)	1 (0.02)	0	1 (0)
Pseudolactococcus raffinolactis	1 (0.01)	1 (0.02)	0	2 (0.01)
Ruminococcus sp.	3 (0.02)	27 (0.64)	4 (0.05)	34 (0.12)
Streptococcus agalactiae	343 (2.12)	3 (0.07)	0 (0)	346 (1.2)

Streptococcus equinus	83 (0.51)	8 (0.19)	1 (0.01)	92 (0.32)
Streptococcus infantarius	4406 (27.19)	405 (9.53)	105 (1.26)	4916 (17.08)
Streptococcus parauberis	0 (0)	0 (0)	1 (0.01)	1 (0)
Streptococcus sp.	37 (0.23)	44 (1.04)	0 (0)	81 (0.28)
Turicibacter sp.	0 (0)	6 (0.14)	0 (0)	6 (0.02)
Vagococcus carniphilus	6479 (39.98)	2575 (60.59)	7542 (90.54)	16596 (57.65)
Veillonella dispar	7 (0.04)	0 (0)	0 (0)	7 (0.02)
Weissella cibaria	2858 (17.64)	220 (5.18)	107 (1.28)	3185 (11.06)
Weissella confusa	2 (0.01)	0 (0)	0 (0)	2 (0.01)
Weissella ghanensis	15 (0.09)	1 (0.02)	1 (0.01)	17 (0.06)
Wolbachia endosymbiont sp.	79 (0.49)	11 (0.26)	50 (0.6)	140 (0.49)
Zymobacter palmae	1 (0.01)	0 (0)	129 (1.55)	130 (0.45)
Total	16206 (100)	4250 (100)	8330 (100)	28786 (100)
